# Travel Trouble With Legionella in the Era of COVID-19: A Case Report

**DOI:** 10.7759/cureus.13632

**Published:** 2021-03-01

**Authors:** Khandakar M Hussain, Md Didar Ul Alam, Nuzhat T Ahmad

**Affiliations:** 1 Internal Medicine, Conemaugh Memorial Medical Center, Johnstown, USA; 2 Internal Medicine, Sylhet MAG Osmani Medical College, Sylhet, BGD

**Keywords:** legionella pneumophila, macrolide resistant legionella, covid 19 overlap syndrome

## Abstract

A 56-year-old male was admitted to the hospital with severe sepsis secondary to pneumonia. His presentation was challenging and confusing due to the accompanying coronavirus disease 2019 (COVID-19) infection attributed to his travel history and diagnosed via radiological findings. He received dexamethasone with ceftriaxone and azithromycin. Despite the fact he was on appropriate antibiotics, his condition worsened, and he was eventually diagnosed with Legionella pneumonia, which was thought to be resistant to macrolides. His condition improved significantly when antibiotics were switched to levofloxacin. It is important to keep in mind other causes of community-acquired pneumonia (CAP) during the ongoing COVID-19 era. What makes this case unique is that it presented a confusing scenario due to the patient's concurrent COVID-19 infection and his failure to improve with the administration of azithromycin.

## Introduction

Legionella bacteria are aerobic, gram-negative, and intracellular pathogens that are major causes of community-acquired and nosocomial pneumonia. Legionnaires' disease accounts for approximately 1-10% of cases of community-acquired pneumonia (CAP) [1-2]. In this report, we present a unique case of severe sepsis secondary to macrolide-resistant Legionella pneumonia that was confused with coronavirus disease 2019 (COVID-19). Signs and symptoms of severe COVID-19 infection and Legionnaires' disease can be similar and may be indistinguishable from each other initially [3]. Failure to identify severe Legionella infection early in the course of the disease may lead to significant mortality and morbidity.

## Case presentation

A 56-year-old male with a past medical history of hypertension and asthma presented to the emergency room with complaints of fever, shortness of breath, and diarrhea for five days. His symptoms had initially started with just fever, and over the course of several days, he had also developed shortness of breath with a productive cough and diarrhea. The patient was a frequent traveler and had been staying in a motel for a few days before the symptoms started. Chest X-ray showed bilateral lung infiltrates. He was in need of 2-L oxygen by nasal cannula on admission. Influenza A, B, and COVID-19 polymerase chain reaction (PCR) testing were negative on admission. Considering his travel history, which made him susceptible to COVID-19 infection, he was started on dexamethasone. He was also given Rocephin and azithromycin. 

**Figure 1:**
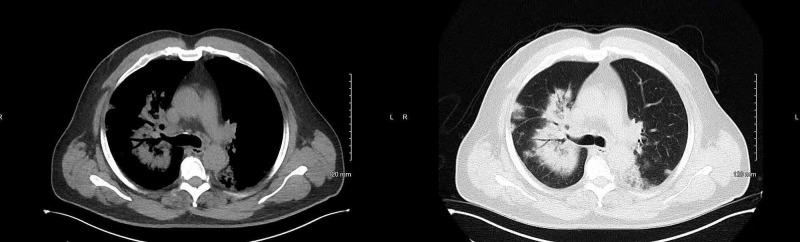
CT chest showing extensive bilateral alveolar infiltrates CT: computed tomography

Initial blood work showed sodium of 131 mmol/l, creatinine of 1.8 mg/dl, blood urea nitrogen (BUN) of 25 mg/dl, mild liver function abnormality with an alanine transaminase level of 88 u/l and aspartate transaminase level of 95 u/l, and a white blood cell count of 15 thousand/uL. The patient was diagnosed with sepsis secondary to multifocal pneumonia. After the admission, his condition started to deteriorate. He became more hypoxic; blood cultures were negative, but he was persistently febrile. Antibiotics were then switched to cefepime and vancomycin, but azithromycin was continued. The patient’s condition deteriorated even further on day two, and he was on 15-L oxygen by high-flow nasal cannula. A CT chest was performed (Figure 1), which showed extensively scattered alveolar infiltrates with air bronchograms throughout both lung fields involving all pulmonary lobes. Taking into account his travel history, COVID-19 PCR testing was done again on day two and day three, which also came back negative. On day three, the Legionella urine test came back positive. The patient was given azithromycin for four days, but his oxygen requirement remained high without significant clinical improvement. Blood cultures remained negative. At that time, it was determined that this could be a case of macrolide-resistant Legionella; his antibiotics were then switched to levofloxacin. Within 24 hours after the antibiotics were switched, his condition improved dramatically. He was eventually discharged on levofloxacin on room air.

## Discussion

At least 26 other Legionella species are pathogenic in humans. Legionella longbeachae (L. longbeachae) is the second most common cause of human diseases. Signs and symptoms of severe COVID-19 infection and Legionnaires' disease can be similar and may be indistinguishable from each other [3]. L. micdadei, L. bozemanii, L. feeleii, L. anisa, and L. dumoffii are also established human pathogens [4-5]. Legionnaires' disease ranges from mild to severe. In a case series of 214 hospitalized patients with Legionnaires' disease, 47% had moderate/severe pneumonia (Pneumonia Severity Index IV to V), and 18% required intensive care unit (ICU) admission [6]. In another study, up to 44% of patients were reported to require ICU admission, and the associated mortality was approximately 1-10% [7]. Older age is strongly associated with Legionnaires' disease. In most cohort studies, more than 75% of the patients with Legionnaires' disease are over 50 years old [8]. Legionellae are environmental organisms found in water and soil. Legionella bacteria are typically transmitted to humans via inhalation of aerosols, derived from water or soil [9-10]. Pneumonia caused by Legionella is clinically and radiographically similar to other forms of pneumonia. They mostly present with the symptoms of fever, cough, and shortness of breath. Symptoms typically emerge 2-10 days after the exposure to contaminated water or soil. Fever and fatigue often precede the onset of cough. Rales and/or other signs of consolidation can be present on physical examination. Radiographic findings are varied and nonspecific; however, the most common findings are patchy unilobar infiltrates, which can progress to consolidations [11-12].

The index of suspicion for Legionella infection should be particularly high during known outbreaks, which are often associated with contamination of water supplies in large facilities such as hospitals, hotels, or apartment buildings. Other epidemiologic factors that should heighten suspicion for Legionella infection include known or potential exposure to a contaminated water source (e.g., hot tubs, birthing pools, fountains) and exposure to soil or potting mix in areas where the incidence of L. longbeachae is high. Immunocompromised patients and severe CAP patients requiring hospitalization should be tested for Legionella [13-14]. PCR on a lower respiratory tract sample (e.g., sputum or bronchoalveolar lavage specimen) has high diagnostic accuracy and detects all Legionella species and serogroups. If PCR is not available or if sputum cannot be obtained, urine antigen testing is an acceptable alternative, especially in regions such as the United States where the prevalence of L. pneumophila serogroup 1 is high. The main advantages of the urinary antigen test are its rapid turnaround time and high specificity. The sensitivity of urine antigen tests ranges from approximately 70 to 80%, and the specificity approaches 100% in patients with Legionnaires' disease caused by L. pneumophila serotype 1 [15,16,17]. Levofloxacin and azithromycin are the preferred agents for the treatment of Legionnaires' disease because these agents are bactericidal, achieve high intracellular concentrations, penetrate lung tissue, and are active against all Legionella species that cause human infection. There are no randomized trials that have directly evaluated the efficacy and side effects of anti-Legionella antibiotics. However, cohort studies have not found much difference in mortality rates when comparing levofloxacin with azithromycin [18,19].

## Conclusions

During the era of COVID-19 infection, it is critically important to rule out other common causes of pneumonia that might have similar presentations. Gathering travel information of such patients is of vital importance. Early diagnosis is crucial as these patients can deteriorate quickly and end up being critically ill. Our patient was getting appropriate treatment with azithromycin. It seems that the Legionella species was resistant to macrolide, as the patient's condition improved dramatically within 24 hours of treatment with levofloxacin. Although there are no randomized data about the efficacy of the two most commonly used antibiotics for Legionella pneumonia, it is important to change the antibiotics if adequate clinical response is not achieved. Our patient improved clinically on levofloxacin. However, there are no randomized trials that have directly evaluated the efficacy of anti-Legionella antibiotics.
